# Informed consent in endoscopy: Read, understood, or just signed?

**DOI:** 10.1016/j.igie.2024.04.001

**Published:** 2024-04-09

**Authors:** Ana Catarina Carvalho, Ricardo Cardoso, Hugo Marcelo Vieira, Américo Silva

**Affiliations:** 1 Department of Gastroenterology, Centro Hospitalar Tondela-Viseu, EPE, Viseu, Portugal; 2 Department of Public Health, Unidade de Saúde Pública ACeS Maia/Valongo, Porto, Portugal

## Abstract

**Background and Aims:**

Although informed consent is a requirement for all interventional procedures such as those in GI endoscopy, its standardization is a challenge. Very thorough documents have been proposed, but it is unknown whether patients actually read them. We evaluated if patients read and understand informed consent forms and information leaflets for GI endoscopy.

**Methods:**

This single-center, prospective, observational study was performed between April 2021 and April 2022 and included adult patients proposed for outpatient elective EGD and colonoscopy. Informed consent forms and information leaflets were mailed to patients, with a small text instruction added to the informed consent form. Before endoscopy, we assessed whether patients adequately read the informed consent form, based on patient signature, table questionnaire completion, and performance of the text instruction.

**Results:**

The study included 232 patients (50.6% men; mean age, 63.8 ± 12.76 years). Most had only a basic education (78.0%) and had previously undergone GI endoscopy (90.6%). Of the patients, 86.6% stated they had read the form and 13.4% did not. Although most signed the form (83.6%), only 24.6% adequately read and understood it. No statistically significant association between an adequate reading of the informed consent form and any of the assessed variables was found.

**Conclusions:**

Despite the timely provision of information, most patients do not read or adequately understand the provided documents. It is necessary to develop new strategies to enhance patients’ involvement in decision-making, thus improving the doctor–patient relationship in obtaining informed consent. (Clinical trial registration number: NCT05414435.)

Informed consent is an ethical and legal process based on the principle that patients have the right to autonomously make an informed choice regarding any medical intervention planned to be performed.^1^^,^^2^ The intent is to provide information and ensure that patients accept the procedure, enhancing the understanding of their own situation and not simply acquiring their signature.^2^ A valid informed consent process requires intention, disclosure, understanding, and the capacity to engage in reasoned deliberation. If any of these elements is lacking, informed consent could be compromised.^3^ This process should always take place before all invasive medical procedures, such as those in GI endoscopy, and must focus on crucial elements according to each patient’s particular situation.^2^^,^^4^ Information given to the patient must include reference to the nature of the proposed procedure; reason it is being suggested; benefits, risks, and potential reasonable alternative interventions; and prognosis if the treatment or examination is declined.^5^

A true informed consent is the cornerstone of good clinical practice, although it is only valid if it reflects a reasonable understanding of the procedure and arises from a nonpressured decision, for which time and opportunity for the patient to ask questions are essential.^4^ Good communication is essential, but several challenges influence adequate informed consent, such as lack of time, concerns about giving too much or too little information, misunderstanding of the information given and an inability to detect a patient’s lack of comprehension, language and cultural issues, or the perception of patients that the document is just a legal release for the physician to proceed.^6^

The obsolete patient–physician relationship in which patients implicitly trust their doctors has been changing over time because information is widely available in the internet era and patients expect choices and become more litigious.^6^ Although this has major implications to the informed consent process, determining how much information is required and guidance about what level of risk must be disclosed is still not clear.^7^^,^^8^ Giving too much information with technical language may restrain or frighten patients and deter them from completely reading or understanding all the information provided; on the other hand, too little information can lead to liability in lawsuits against healthcare providers, considering the patient was not diligently informed about all risks.^2^ Failure to adequately inform patients is considered negligence and a determining factor for liability claims against physicians.^9^

Regarding GI endoscopy, although the way in which to obtain informed consent has not been standardized, information provided during informed consent should be given as a document written in lay language that patients find comprehensible and require a signature from both patient and physician, confirming their consent to perform the endoscopic procedure.^1^^,^^10^ To tackle some of these challenges, the Portuguese Society of Digestive Endoscopy (SPED) recently developed proposals for informed consent forms and information leaflets for EGD and colonoscopy.^11^, ^12^, ^13^, ^14^

Literacy issues are still common in the general population, and there is a widespread opinion that patients still do not adequately read information provided by healthcare providers; however, a few studies have assessed the readability and understanding of written informed consent forms.^15^^,^^16^ The aim of this study was to evaluate if patients read and understand informed consent forms for GI endoscopy.

## Methods

### Study design and patient selection

This single-center, prospective, observational study was performed between April 2021 and April 2022 in a secondary nonacademic hospital. The reporting of this study conforms to the Strengthening the Reporting of Observational Studies in Epidemiology statement for cohort studies.^17^

We included patients aged ≥18 years who were able to autonomously give their informed consent and were referred for outpatient elective GI endoscopy (diagnostic and/or therapeutic upper GI [UGI] endoscopy and/or colonoscopy) without deep sedation between April 2021 and April 2022. Patients referred for urgent endoscopic procedures (UGI endoscopy and/or colonoscopy) or with deep sedation were excluded.

Patients were consecutively selected to participate in the study and mailed a letter at least 3 weeks before the endoscopic procedure, containing both information leaflets about the endoscopic procedure and an informed consent form proposed by SPED as well as instructions attached to direct patients to read carefully and bring back the documents at the day of the procedure (Appendices A-D, available online at www.igiejournal.org). The SPED informed consent form was modified to incorporate a simple small text instruction at the end of the document, stating “If you have read the information up to this point, please underline this sentence.”

As the standard procedure in our institution, all patients referred for endoscopy had already consented to the procedure and signed a proprietary consent form before endoscopy. The consent for the scheduled endoscopy was considered valid if patient and physician signatures were present in the proprietary form and was reconfirmed at the time of the procedure, regardless of whether they signed the SPED consent form sent in the scope of this study.

Immediately before the endoscopic procedure, all selected patients were informed about the study and were asked whether they would be interested in participating. All interested patients had the opportunity to ask questions and signed a research consent form before participating in the study. Patients who decided to participate were asked to respond to a small questionnaire (Appendix E, available online at www.igiejournal.org). The questionnaire consisted of questions about patient demographics and the following questions:

1. What kind of procedure are you undergoing?
2. Have your ever undergone an endoscopic procedure? If yes, was it in our center?
3. Did you read the informed consent document prior to the procedure?
4. Why did you not read the informed consent form?
5. Did you have any doubts after reading the informed consent form?
6. Did you change your opinion regarding the procedure after reading the informed consent form?

It was then assessed whether patients adequately read the informed consent form, based on 3 criteria. Adequate reading was considered only if patients fulfilled all of the following:

1. Signature of the form
2. Filling in the table questionnaire regarding medical history and medication (any single answer was considered toward adequate reading, independently from accuracy or completeness of the reply)
3. Performance of the text instruction added to the original version of the consent form

Anonymity of all patients was guaranteed by assigning a sequential number to the questionnaires that did not include identifying data.

### Data collection

Data collected were age, sex, education level, endoscopic procedure performed, if conscious sedation was performed, previous endoscopic procedures, and place of previous endoscopic procedure. All patients’ details were deidentified.

### Statistical analysis

The collected data were analyzed with SPSS (version 28.0; IBM SPSS Statistics for Windows, Armonk, NY, USA). Descriptive statistics were used for patient demographics and disease characteristics. Categorical variables are given as proportions or absolute count, and comparisons were made with the Pearson χ^2^ test and Fisher exact test. Continuous data are expressed as mean and standard deviation for normally distributed data or median and interquartile range for non-normally distributed data; comparisons were performed with the Student *t* test and analysis of variance tests or Mann-Whitney and Kruskal-Wallis tests. Results are reported as odds ratio with 95% confidence intervals. Factors that had a potential to correlate with patients’ reading of the informed consent form for endoscopic procedures were subjected to multivariate analysis with the logistic model. All *P* values were 2-tailed, and *P* < .05 were considered statistically significant.

### Ethical considerations

The study was approved by the local ethics committee (Ethics Committee of Centro Hospitalar Tondela-Viseu, E.P.E, November 25, 2019) and conducted according to the Declaration of Helsinki. The study was registered in clinicaltrials.gov (NCT05414435).

### Results

**Figure 1:**
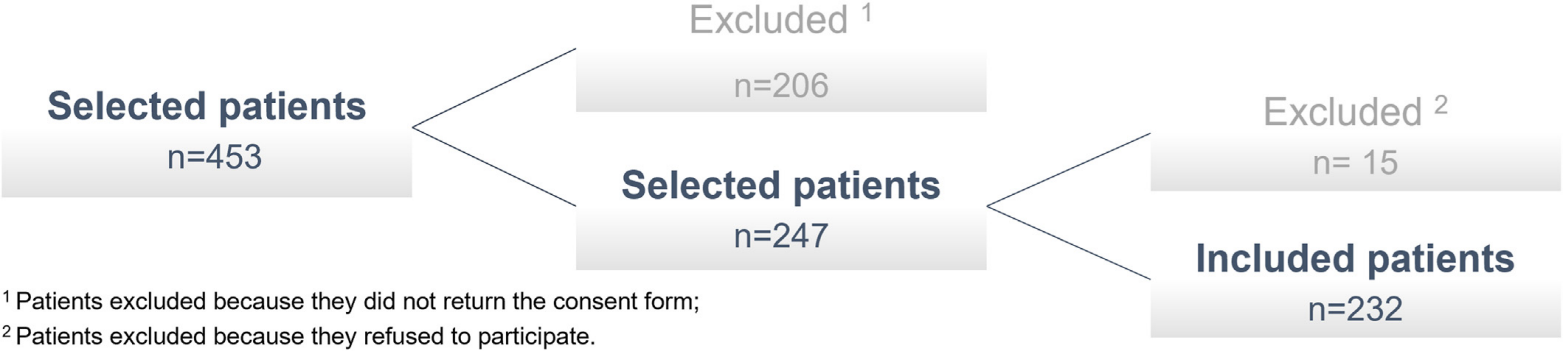
Selection of patients in this study.

Between April 2021 and April 2022, 453 patients were selected to participate in this prospective observational study. Of these, only 247 patients (54.5%) brought back the consent form on the scheduled day of the procedure. In total, 221 patients were excluded because they did not return the consent form after their attendance (n = 206) or refused to participate (n = 15) (Fig. 1). The study included 232 patients, 130 men (56.0%) and 102 women (44.0%), with a mean age of 63.8 ± 12.7 years. Sociodemographic data of enrolled patients are described in Table 1.

**Table 1:** Sociodemographic characteristics of the study population

Characteristics	Values
Age, y	63.8 ± 12.8
Sex	
Male	130 (56.0)
Female	102 (44.0)
Education	
Illiterate	7 (3.0)
Basic education	181 (78.0)
Superior education	44 (19.0)
Endoscopic examination and sedation	
Upper GI endoscopy	110 (47.4)
With conscious sedation	23 (9.9)
Without conscious sedation	87 (37.5)
Colonoscopy	107 (46.1)
With conscious sedation	10 (4.3)
Without conscious sedation	97 (41.8)
Upper GI endoscopy + colonoscopy	15 (6.5)
With conscious sedation	1 (.4)
Without conscious sedation	14 (6.0)
Previous endoscopic examination	
Yes	211 (90.9)
No	21 (9.1)

Value are mean **±** standard deviation or n (%).

Most patients (78.0%) had only a basic education (<9 years of education), and 3.0% of them were illiterate and did not know how to read or write. UGI endoscopy was the endoscopic procedure performed in most patients (47.4%), and only 6.5% underwent both UGI and colonoscopy at the same appointment. Most endoscopic examinations (85.3%) were performed without the patient under conscious sedation (procedures with patient under deep sedation were excluded from this study, as stated above). Most patients (90.9%) had previously undergone GI endoscopy (either UGI endoscopy or colonoscopy), mostly in our gastroenterology department.

Patient perception of informed consent forms and information leaflets are described in Table 2. When patients were asked if they had read the documents regarding the endoscopic procedure that were mailed, 201 patients (86.6%) stated they had read the informed consent form and 31 (13.4%) did not. The reasons patients gave for not reading the documents included the following: “Had no time or interest in reading the document” (n = 22, 71.0%), “I don’t know how to read or write” (n = 4, 12.9%), “My family read it for me” (n = 3, 9.7%), or “The letter size is too small for me” (n = 2, 6.5%). None of the patients changed their mind about undergoing the endoscopic examination after reading the informed consent form. However, only 7 patients (3.0%) asked questions and requested more information regarding risks and adverse events and alternative methods to the endoscopic procedure.

**Table 2:** Patient perception of informed consent form and information leaflet

Questions	No. answered “yes” (%)
Did you read the informed consent form before the procedure?	201 (86.6)
Why you did not read the informed consent form?	
“Had no time or interest in reading the document”	22 (71.0)
“I don’t know how to read or write”	4 (12.9)
“My family read it for me”	3 (9.7)
“The letter size is too small for me”	2 (6.5)
Did you have any doubts after reading the informed consent form?	7 (3.0)
Did you change your opinion regarding the procedure after reading the informed consent form?	0

**Figure 2:**
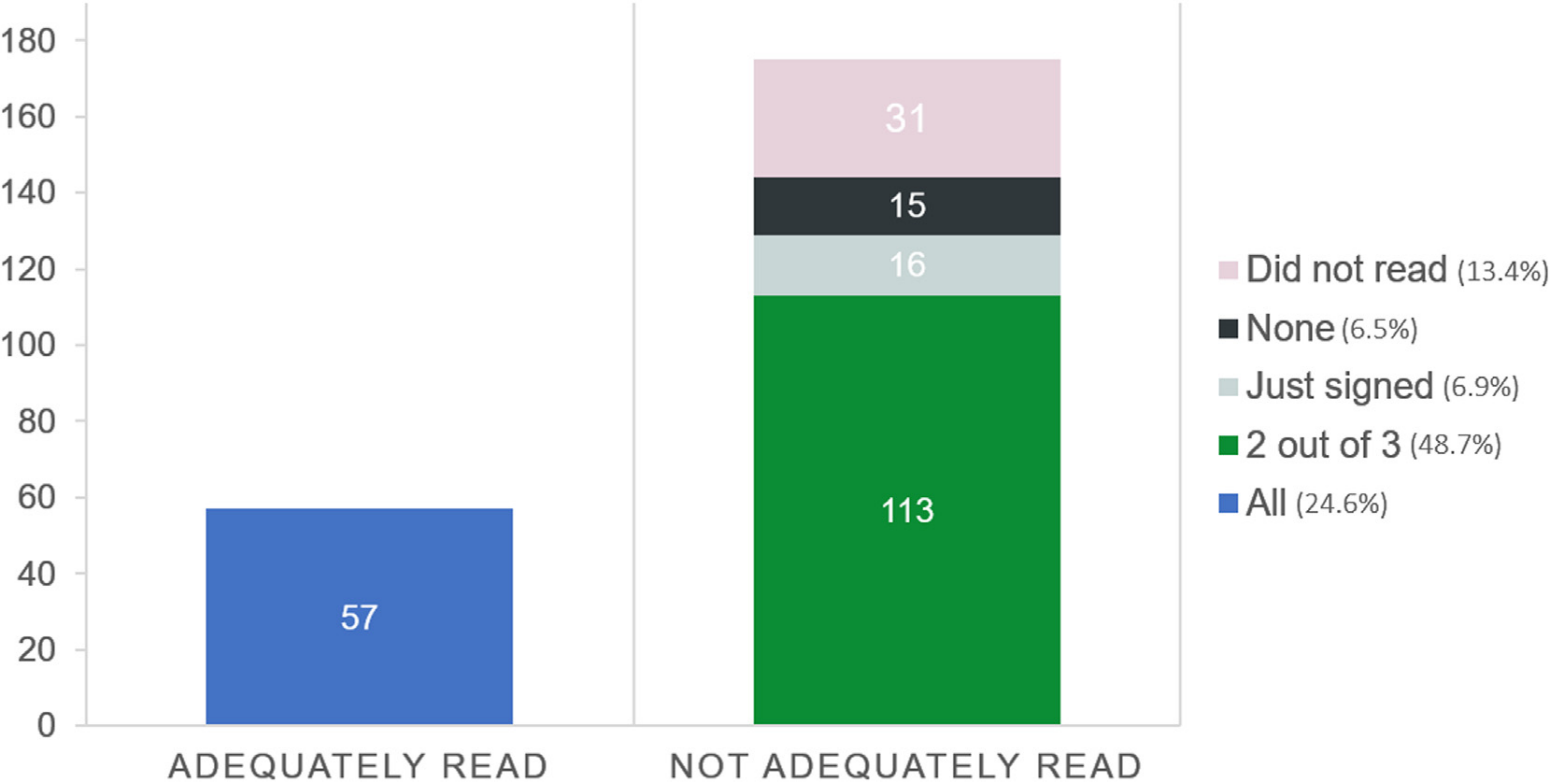
Number of patients that fulfilled each criteria for adequate reading.

Although 180 patients (77.6%) signed the form, only 57 (24.6%) adequately read and understood the document. The table questionnaire was completed by 72.0% of patients, the text instruction was performed by 26.3%, 16 patients (6.9%) just signed the form, and 15 patients (6.5%) did not fulfill any criteria (Fig. 2). Although 31 patients stated they had not read the document, 14 (6.0%) of them signed the consent form anyway.

Table 3 shows the factors affecting patients’ reading of the forms. Factors with the potential to correlate with an adequate reading of the informed consent form, namely age, sex, education level, type of endoscopic procedure, or sedation, were subjected to multivariate analysis. Patients aged ≥65 years seemed to read the informed consent forms more thoroughly when compared with younger patients (odds ratio, 1.989; *P* = .027). Although the percentage of female and more educated patients who adequately read the informed consent seemed to be higher, no statistically significant difference was found regarding other variables such as education grade, sex, or type of endoscopic procedure performed.

**Table 3:** Multivariate analysis of factors related to patient reading of informed consent forms

	Adequately read (n = 57)	Not adequately read (n = 175)	Odds ratio (95% confidence interval)	Adjusted odds ratio (95% confidence interval)∗	*P* value
Age, y	65.2 ± 14.2	63.4 ± 12.2	1.012 (.988-1.037)	—	.332
Age					
<65 y	21 (18.3)	94 (81.7)	1.00	1.00	.027
≥65 y	36 (30.8)	81 (69.2)	1.989 (1.076-3.679)	1.989 (1.076-3.679)	
Sex					
Female	29 (28.4)	73 (71.6)	1.00	—	.226
Male	28 (21.5)	102 (78.5)	.691 (.379-1.259)	—	
Education					
Illiterate/basic	45 (23.9)	143 (76.1)	1.00	—	.644
Superior	12 (27.3)	32 (72.7)	1.192 (.567-2.506)	—	
Endoscopic examination					
Upper GI endoscopy	31 (28.2)	79 (71.8)	1.00	—	
Colonoscopy	25 (23.4)	82 (76.6)	.777 (.422-1.431)	—	.418
Both	1 (6.7)	14 (93.3)	.182 (.023-1.444)	—	.107
Conscious sedation					
No	47 (23.7)	151 (76.3)	1.00	—	.478
Yes	10 (29.4)	24 (70.6)	1.339 (.597-3.000)	—	
Previous examination					
No	4 (19.0)	17 (81.0)	1.00	—	.538
Yes	53 (25.1)	158 (74.9)	1.426 (.459-4.425)	—	

Value are mean ± standard deviation or n (%). —, not applicable.

∗ The adjusted odds ratio was calculated using multivariate logistic regression, including sex, age, and all variables with odds ratio with *P* ≤ .1, using a backward stepwise regression.

## Discussion

In this prospective observational study, we assessed if patients read and understand informed consent forms developed for GI endoscopy. Considering that technologic progress has enriched gastroenterology with new and more invasive endoscopic capabilities, concerns about legal issues are also increasing as well as the need for guidelines on how to establish an informed consent.^9^ In this regard, as recommended by the British Society of Gastroenterology and American Society for Gastrointestinal Endoscopy guidelines for informed consent, all endoscopic departments should demand written documentation of informed consent.^2^^,^^18^ However, standardization of consent forms is a challenge because some demands are difficult to meet, such as the extensive documentation of specific factors that could increase the adverse event risk of a given individual. Therefore, it is debatable if generic and understandable information should be provided in contrast to a detailed and extensive version that includes all risks, which could discourage the patient from undergoing the endoscopy.^19^^,^^20^ Although British Society of Gastroenterology guidelines recommend that “if an individual’s risk is higher owing to frailty or comorbidity, that should be discussed and/or provided additional written information to reflect this risk and that information documented in the case notes,” the depth of information patients require to deliberate and consent to an invasive procedure is very variable.^18^^,^^21^

A study performed to determine the levels of information required by patients and solicitors specializing in medical malpractice revealed that 16% of solicitors and 6% of patients believed that informed consent forms should describe very uncommon risks such as those with odds of 1 in 1,000,000.^7^ On the other hand, a study performed to assess the impact of postal information packets on the understanding of consenting for GI endoscopy revealed that 10.8% of patients were not bothered if the risk was rare (<1 in 1000).^22^ In our study, a high percentage of patients (45.5%) did not bring back the informed consent form as stated in the mailed letter at the scheduled day of the endoscopic procedure, suggesting a misconceived perception that informed consent would not be necessary to perform endoscopic examinations or a lack of awareness that endoscopic procedures are invasive procedures with non-negligible risks. Also, the fact that in our study over 75% of patients signed the informed consent form but less than 25% adequately read it emphasizes the misconception that “informed consent” is a matter of signing a form and not a process of disclosure and deliberation about the procedure. A similar study performed in a Veterans Administration population that included 59 male patients scheduled for screening sigmoidoscopy revealed that although all patients signed the consent form, only 14% of patients actually read it completely.^23^

In this study, only elective procedures on outpatients were included because informed consent in inpatients happens in a different scenario and can be waived in emergent procedures.^2^ Although all invasive procedures require informed consent, hospitalized patients are compromised by their illness and may believe they are impaired to autonomously decide. Conversely, outpatients undergoing elective endoscopic procedures have adequate time to acknowledge, understand, and decide about what they may undergo. Also, informed consent should not be performed just before undergoing invasive procedures, because patients are required to assimilate a lot of new and complex information and may find it difficult to make a thoughtful decision.^18^^,^^24^ It has been reported that when information is given about 1 to 2 weeks before a procedure, nearly 94% of patients read information leaflets and 92% sign informed consent forms. In contrast, if information is given immediately before the procedure, 46% of patients sign the document without reading it.^24^^,^^25^ In our study, both informed leaflets and informed consent forms were sent in advance of the scheduled endoscopic procedures by 3 to 4 weeks, thus allowing patients reasonable reflection time to understand and fully deliberate on the procedure to which they were consenting. However, only 24.6% of patients adequately read and understood the informed consent form, demonstrating that despite timely provision of information, most patients do not adequately read these documents.

Another challenge for a truly informed consent is the influence of sociodemographic factors such as sex, age, and education in reading and comprehension achievement. In this regard, younger age and higher education have been found to correlate with a better capability to store and recall information, whereas difficulties are more glaring in patients aged ≥40 years, those with lower levels of education, and nonwhites.^9^^,^^26^^,^^27^ In contrast to published data,^1^ in our study patients aged >65 years seemed to adequately read the informed consent form. However, the proportion of patients aged <40 years in our study population was small (4.7%), which may explain this finding. Regarding education level, in contrast to similar studies, no relationship was found in our study between proper reading of consent forms and degree of education, even though the number of elderly patients, who are most likely to only have basic education, was higher than younger patients.^9^^,^^28^ As far as sex is concerned, as described in similar studies assessing readability of consent forms, our results indicate that adequate reading does not seem to be related to this sociodemographic variable.^15^^,^^29^ In conclusion, apart from age, our results are independent of any assessed sociodemographic variables, similarly to the study performed in a Veterans Administration population mentioned above,^23^ in which the percentage of patients who actually read the consent form (14%) was also independent of ethnicity, educational level, and previous sigmoidoscopy experience.

Besides sociodemographic variables, other factors could theoretically contribute to more attention in reading consent forms, such as type of endoscopic procedure, mainly colonoscopy given its better known risk of perforation, and previous experience in GI endoscopy or sedation, which could increase procedure-specific anxiety in those who had never undergone endoscopy before. However, none of these variables seemed to be related to a better reading of informed consent forms in our study. These findings could be explained by the small sample size of our study, the fact that elective procedures are perceived to be safe and patients do not expect an adverse event to occur, or the idea that informed consent forms are a waste of time, as stated by 71% of patients who stated they did not read the documents.

Although often overlooked, sentence and word length, as well as font size and style, may affect both the readability and comprehensibility of the text. These factors are assessed using readability formulas such as the Flesch Reading Ease Formula, Gunning Fog Scale, Automated Readability Index, and the Coleman-Liau Index, among others.^30^ Published data recommend that patient information should be aimed at a sixth-grade level (10-11 years old) because of a significant prevalence of literacy issues even in Western countries.^31^ Using the Flesch Reading Ease Formula, we concluded that the level of readability of SPED informed consent forms and information leaflets is very difficult to read, which means the level of readability of this text is above the comprehension level of our study sample and that patients needed to receive a college graduate education to easily understand the documents. A study performed to assess the readability of informed consent forms before anesthesia concluded that these forms are not appropriate for individuals who received less than 8 years of education.^32^ Although in Portugal 31% of the population between ages 25 and 64 years have a higher education, in our study only 19.0% of patients had a higher education.^33^ Although we did not find any correlation between education level and adequate reading, the high percentage of patients with only a basic education in our study combined with the difficulty of the informed consent forms according to the Flesch Reading Ease score may explain why patients did not adequately read the documents. Furthermore, it is plausible that the complexity of the text by itself and independently of patient education level may have reduced the willingness to read and pay adequate attention to the entire document. This emphasizes the need to develop written documents for patients with medical information provided in simpler and easy to understand text to make them understandable to individuals in all levels of education.^15^

Our study has several limitations, mostly because it was from a single center with a small sample size and suboptimal subject participation. The exclusion of outpatients referred for elective endoscopic procedures with deep sedation further limited our sample size. However, these patients are usually evaluated by an anesthesiologist at a scheduled appointment before the endoscopic procedure, and deep sedation also requires an informed consent process and signing a written form, different from that performed by the endoscopist. Therefore, these patients were excluded because of a potential bias concerning confusion about which informed consent form they were asked if they had read.

It may also be argued that comprehension was not truly evaluated. However, confirmation of a complete grasp of medical information is very hard to define and evaluate, and we believe the closest available surrogate is the compliance with text instructions. Although the artifice that was created to test if the patients actually read the entire document is not validated, we are not aware of an adequate alternative in the literature. Other methods such as a multiple-choice test were rejected because they would further increase complexity and cumbersomeness.

Another possible bias is the phenomenon often called the “Hawthorne effect” or “trial effect” in which the subjects of a clinical trial change their behavior because they are aware of being studied, potentially affecting results, namely by increasing compliance.^34^ However, we believe this effect was mitigated because patients were invited to participate in the study immediately before the procedure, long after the form was provided, completed, and collected. Furthermore, the expected direction of the skewing would be a higher number of patients reading and completing the instructions, whereas the opposite was observed.

Although we did not find a significant correlation between patients undergoing previous endoscopic procedures and adequate reading of the consent form and informative leaflets, only 9.1% of patients had no experience regarding GI endoscopy. These results could be different if patients were referred for endoscopy for the first time in a community-based endoscopic units.

Finally, it could be suggested that the use of 2 consent documents, the proprietary form and the altered study document, could potentially confuse patients and reduce the rates of participation (negatively impacting the sample size) and willingness to read the entire document, thus affecting the primary endpoint. However, a simple substitution of the proprietary form with the new one would not be acceptable because the former is the approved and legal-binding document in the institution. To mitigate this bias, the letter sent to the patients clearly stated, in the cover, that the accompanying documents should be carefully read, completed, and brought back at the day of the procedure.

In conclusion, in the first Portuguese study that assessed if patients truly read and understand informed consent forms in GI endoscopy, we found that despite timely provision of information most patients do not, and signing a form does not mean consent was genuinely informed. Although these findings may not be directly applicable to other settings because of several differences such as sociodemographic background, health systems, sedation practices, and the specific informed consent forms used, they raise the question whether our patients really understand to what they are consenting.

Our results emphasize the need for educational interventions to highlight the importance of informed consent as an active informed decision and to improve communication in clinical practice, which is much needed in obtaining a true informed consent. Strategies to improve readability such as shorter sentences and replacing jargon with simpler layperson terms or combining written information with infographics or even short videos are likely to be most effective and useful to patient understanding and decision-making, although they are no substitute for a verbal discussion of information that is crucial in an effective doctor–patient interaction, either in person or virtually, such as in a phone call ahead of time.^8^^,^^35^

## Disclosure

All authors disclosed no financial relationships.

## Supplemetary data

Appendix A Informed consent form for EGD and upper GI endoscopy proposed by the Portuguese Society of Digestive Endoscopy (modified).

Appendix B Information leaflet for EGD. Additional information regarding upper GI endoscopy with or without sedation (including biopsy sampling and polyp removal as needed).

Appendix C Informed consent form for colonoscopy proposed by the Portuguese Society of Digestive Endoscopy (modified).

Appendix D Information leaflet for colonoscopy. Additional information regarding colonoscopy with or without sedation (including biopsy sampling and polyp removal as needed).

Appendix E Questionnaire on informed consent for endoscopic procedures applied before the endoscopic procedures.
