# Supplementary material for: Informed consent in endoscopy: Read, understood, or just signed?
Source: IGIE. 2024 Apr 9;3(2):222–9. doi: 10.1016/j.igie.2024.04.001 (PMC12850725; doi:10.1016/j.igie.2024.04.001)
Supplement: Appendix A — Informed consent form for EGD and upper GI endoscopy proposed by the Portuguese Society of Digestive Endoscopy (modified). [file mmc1.pdf]

Upper Gastrointestinal Endoscopy with or without sedation (including biopsies and polyp removal as needed) - Free and informed consent

Please, read this entire document carefully as this information is very important. Together with this consent, you will receive a second document with additional information on this procedure.

Upper gastrointestinal endoscopy is a procedure that allows the observation of the oesophagus, stomach and the initial duodenum, by means of a flexible device named endoscope (approximately 10 mm in diameter), which is equipped with a small camera. In general, upper gastrointestinal endoscopy is a safe procedure with a complication rate of less than 0.2%. Still, adverse events may occur during diagnostic and/or therapeutic exams.

The most common adverse events include:

- Pain or mild discomfort at the neck, thorax or abdomen;
- Transient vomiting and/or swallowing difficulty;
- Dizziness or fainting sensation after standing at the end of the procedure;
- Headache;
- Pain, redness, and swelling at the puncture site where sedation or medication was administered (in case sedation/medication was administered);
- Muscle pain;
- Allergic reaction to drugs that were administered during the procedure.

Severe complications associated to this procedure are rare, and include:

- **Cardiovascular and respiratory complications**, such as severe allergic reactions called anaphylaxis, "heart-attack", pulmonary embolism, cardiac arrythmia, stroke, and aspiration of food/fluids into the lungs leading to pneumonia. Though rare, these events are more common during exams where sedation was administered, emergency exams, and for elderly people with other conditions (anaemia, dementia, pulmonary disease, obesity, cardiac insufficiency, valvular heart disease);
- **Haemorrhage**, especially if additional procedures are performed (biopsies, polypectomies, dilation…), and if the patient is taking anticoagulant/antiplatelet medication or has blood clotting disturbances;
- **Laceration or perforation** of the oesophagus, stomach or duodenum (rare for merely diagnostic exams - 0.03% risk), especially if additional procedures are performed (biopsies, polyp removal, dilation…);
- **Meta-hemoglobinemia**, which translates into blood oxygenation imbalance. This complication is more common during exams where topical anaesthetic is used (especially benzocaine);
- Other extremely rare complications, namely spleen rupture, large abdominal vessel lesions (mesenteric vessels), diverticulitis (diverticula inflammation) and appendicitis (ileocecal appendix inflammation).

Should these complications occur, in most cases they are managed and solved during the endoscopic procedure. However, in certain cases, adequate and definitive complication treatment may require blood transfusion, surgical intervention and hospitalization. If the procedure is scheduled with sedation/anaesthesia, the patient will be monitored throughout the exam. Sedation-related complications may occur as previously discriminated.

As in other invasive health-related interventions, there is a risk of mortality for both therapeutic and diagnostic procedures, though this risk is extremely low.

Upper gastrointestinal endoscopy is not a foolproof examination and there is a possibility that some lesions may not be detected (example: the rate of tests not revealing a gastric cancer that already exists can reach 14%). This risk is higher if there is residual content in the stomach or if the patient's tolerance is limited. Therefore, a final diagnosis may not be 100% guaranteed.

Do not hesitate to ask for additional information, and to question the clinical team who requested the exam or is going to perform the procedure – this is a right that assists you.

It is essential that you inform the clinical team and the performing physician about your medical history and the medication you usually take. Please pay special attention to the following chart. You must complete this chart with the utmost rigor so that risks are not increased during the procedure.

Upper Gastrointestinal Endoscopy with or without sedation (including biopsies and polyp removal as needed) - Free and informed consent

Please fill in the following table. This is mandatory for the procedure to be performed.

**Please name all the medication you usually take:**		

**Mark with an (X), according to your previous medical history:**		
Previous surgeries?	Yes ( )	No ( )
Removal of the oesophagus or stomach?	Yes ( )	No ( )
Surgeries to the thorax or abdomen?	Yes ( )	No ( )
If yes, please specify:		
History of diverticula (pouches) in the oesophagus or intestine?	Yes ( )	No ( )
Chronic lung disease?	Yes ( )	No ( )
Heart disease?	Yes ( )	No ( )
If yes, please specify:		
Pacemaker/defibrillator?	Yes ( )	No ( )
Artificial cardiac valves?	Yes ( )	No ( )
Known medication allergies	Yes ( )	No ( )
If yes, please specify:		
Latex allergy?	Yes ( )	No ( )
Liver cirrhosis?	Yes ( )	No ( )
Blood clotting diseases?	Yes ( )	No ( )
Diabetes mellitus?	Yes ( )	No ( )
Renal disease?	Yes ( )	No ( )
Possible pregnancy?	Yes ( )	No ( )

Please read this entire document carefully, as well as the additional detailed information document that was handed to you. **If you have read the information up to this point, please underline this sentence.** Check that all the information you provided is correct. The doctor performing the procedure will confirm that you are appropriately informed for the procedure to be carried out. If you have no further concerns, please sign the statement below:

I declare that I have been given an informative document, and that I realized the advantages, risks and complications that may be associated with this examination/diagnostic and/or therapeutic intervention (UPPER GASTROINTESTINAL ENDOSCOPY), namely the risk of perforation, haemorrhage, cardiorespiratory complications, including the risk of death, and that I authorize not only its execution, but also the associated procedures and medical acts necessary to resolve possible complications. I have been provided with the information and clarification I considered necessary. I know that I have the right to change my mind by revoking my consent even after signing this document, but I must immediately make it known to the clinical team.

Patient's full name: ____

Date: _____/_____/_____ | Signature of the patient or his/her guardian: _________________________

DECLARATION

Here in, I confirm that the patient indicated above received the essential information to be adequately informed regarding the intervention that is referred in this document. Additionally, I was available to answer all the questions raised before the procedure.

Physician's full name: ____

Date: _____/_____/_____ | Signature of the performing physician: ______________________________
