# Supplementary material for: Informed consent in endoscopy: Read, understood, or just signed?
Source: IGIE. 2024 Apr 9;3(2):222–9. doi: 10.1016/j.igie.2024.04.001 (PMC12850725; doi:10.1016/j.igie.2024.04.001)
Supplement: Appendix B — Information leaflet for EGD. Additional information regarding upper GI endoscopy with or without sedation (including biopsy sampling and polyp removal as needed). [file mmc2.pdf]

Upper Gastrointestinal Endoscopy with or without sedation (including biopsies and polyp removal as needed) – Additional information

Please, read this entire document carefully as this information is very important.
This document complements and details the informed consent form

Upper gastrointestinal endoscopy is a procedure that allows the observation of the oesophagus, stomach and the initial duodenum. A flexible device named endoscope (approximately 10 mm in diameter), which is equipped with a small camera that transmits the enlarged images to a monitor within the procedure room, will be introduced through the mouth and gently pushed through the oesophagus, stomach and duodenum.
During the procedure, the patient will be lying on its left side. To allow the mouth to remain open during the examination, a plastic mouth guard will be positioned between the patient's teeth. The endoscope passes through this mouth guard. If the procedure is scheduled without sedation, when the endoscope is advanced through the mouth and throat you may be asked to swallow and you may feel transient vomiting sensation and shortness of breath, without pain. Throughout the examination, the patient may breathe normally. Also, the patient may be able to make sounds, but will not be able to speak. To allow the distension and adequate observation of the upper gastrointestinal tract, some air insufflation is necessary, being responsible for some sensation of gastric distension and fullness.

During the procedure, biopsies (small fragments of tissue obtained by a biopsy forceps that runs through the endoscope) may be required for analysis to complement diagnosis. Also, other instruments may be introduced through the endoscope to allow diagnostic and/or therapeutic procedures, such as polypectomies (polyps removal with a forceps or snare), endoscopic drug injection, lesion tattooing, and application of therapy/devices aiming for bleeding prevention/control (clips – small pieces of metal; endoloops – ties). Some of these interventions have additional costs and can be charged after the procedure. Please check this with the health institution where the procedure will take place.
When the examination is complete, the endoscope is slowly removed through the mouth. An upper gastrointestinal endoscopy lasts approximately 5 minutes, but it may be shorter or longer depending on the patient's tolerance, the exam's indication, and the need to perform therapeutic procedures.
In some cases, intravenous sedation may be administered, and this will significantly decrease the procedure-related discomfort. Also, an anaesthetic spray may be applied to the mouth and throat to decrease local sensitivity to the endoscope passage.
After an endoscopy without intravenous sedation, the recovery is very fast (some minutes). In case sedation is performed, after the procedure the patient will be kept in a surveillance room for about 60 minutes to recover from this sedation.
On the procedure day, patients may report discomfort due to gas accumulation in the stomach and bowel (flatulence, abdominal cramps), and discomfort at the throat level. Both will improve over time.

In what situations is an upper gastrointestinal endoscopy indicated?
The recommendation to perform any examination is always made by a Physician according to the patient's characteristics, complaints and diseases. Also, the decision to perform additional therapeutic interventions will depend on the patient's clinical situation.

Upper gastrointestinal endoscopy may be recommended in the following situations:

1. Symptom investigation: nausea, vomiting, abdominal pain, swallowing difficulties, digestive bleeding;
2. Diagnosis: anaemia and diarrhoea causes, inflamed mucosa biopsies, tumour detection;
3. To clarify previous endoscopic findings, or doubts raised in different exams (oesophagus, stomach and duodenum radiographies, abdominal or thoracic computed tomography, ultrasounds or blood results);
4. Treatment: although upper gastrointestinal endoscopy is generally a diagnostic procedure, it may also be therapeutic and curative allowing for esophageal dilation, removal of foreign bodies, polyps removal, and application of therapy aiming for bleeding prevention (endoscopic drug injection, application of thermal energy on anomalous vessels…).

This procedure is invasive and there are risks associated with this exam. These risks may be increased if additional interventions are needed during the exam. When your Physician requested the procedure, its details, aims and risks should have been explained to you. However, it matters to state that not taking an upper gastrointestinal endoscopy that was recommended by a Physician may be associated with the

absence of diagnosis and/or treatment of lesions and pathologies affecting the oesophagus, the stomach and the duodenum (example: stomach cancer).

In general, upper gastrointestinal endoscopy is a safe procedure with a complication rate of less than 0.2%. Still, adverse events may occur during diagnostic and/or therapeutic exams.

The most common adverse events include:

- Pain or mid discomfort at the neck, thorax or abdomen;
- Transient vomiting and/or swallowing difficulty;
- Dizziness or fainting sensation after standing at the end of the procedure;
- Headache;
- Pain, redness, and swelling at the puncture site where sedation or medication was administrated (in case sedation/medication was administered);
- Muscle pain;
- Allergic reaction to drugs that were administrated during the procedure.

Severe complications associated to this procedure are very rare, and include:

- **Cardiovascular and respiratory complications**, such as severe allergic reactions called anaphylaxis, “heart-attack”, pulmonary embolism, cardiac arrythmia, stroke, and aspiration of food/fluids into the lungs leading to pneumonia. Though rare, these events are more common during exams where sedation was administered, emergency exams, and for elderly people with other conditions (anaemia, dementia, pulmonary disease, obesity, cardiac insufficiency, valvular heart disease);
- **Haemorrhage**, especially if additional procedures are performed (biopsies, polypectomies, dilation…), and if the patient is taking anticoagulant/antiplatelet medication or has blood clotting disturbances;
- **Laceration or perforation** of the oesophagus, stomach or duodenum (rare for merely diagnostic exams - 0.03% risk), especially if additional procedures are performed (biopsies, polyp removal, dilation…);
- **Meta-hemoglobinemia**, which translates into blood oxygenation imbalance. This complication is more common during exams where topical anaesthetic is used (especially benzocaine);
- Other extremely rare complications, namely spleen rupture, large abdominal vessel lesions (mesenteric vessels), diverticulitis (diverticula inflammation) and appendicitis (ileocecal appendix inflammation).

Should these complications occur, in most cases they are managed and solved during the endoscopic procedure. However, in certain cases, adequate and definitive complication treatment may require blood transfusion, surgical intervention and hospitalization.

As in other invasive health-related interventions, there is a risk of mortality for both therapeutic and diagnostic procedures, though this risk is extremely low.

Upper gastrointestinal endoscopy is not a foolproof examination and there is a possibility that some lesions may not be detected (example: the rate of tests not revealing a gastric cancer that already exists can reach 14%). This risk is higher if there is residual content in the stomach or if the patient’s tolerance is limited. Therefore, **a final diagnosis may not be 100% guaranteed.**

If the procedure is scheduled with sedation/anaesthesia, the patient will be monitored throughout the exam. Sedation-related complications may occur as previously discriminated.

Do not hesitate to ask for additional information. You will be given the opportunity to speak with the performing Physician and with the Anaesthesiologist (in case your exam is scheduled under deep sedation performed by an Anaesthesiologist) before the endoscopy is carried out.

Upper Gastrointestinal Endoscopy with or without sedation (including biopsies and polyp removal as needed) – Additional information

Additional recommendations:

1. A six-hour fast is mandatory to perform the procedure, which means the patient must not eat solid food nor drink in the 6 hours before the exam. Do strictly comply with this recommendation. If you are not fasting let the medical team know prior to the procedure as serious complications may occur during the exam if patients are not fasting.
2. In case sedation is performed, after the procedure the patient will be discharged but may only leave accompanied, as driving, machine-handling and other high responsibility activities are not allowed for the rest of the day. Also, within 12-24h after the exam, the patient must not sign legal binding documents. If the patient is not accompanied, the procedure must not be performed with sedation and in some cases, it may even be cancelled.
3. The patient must bring a record of all medication he/she usually takes and write it down in the informed consent table where this is requested. This is especially important if the patient is taking anticoagulants: warfarin (Varfine®), acenocumarol (Sintron®), Pradaxa®, Xarelto®, Eliquis®, Lixiana®; and antiplatelets: acetylsalicylic acid (Aspirina®, AAS®, Cartia®, Tromalyt®), ticlopidine (Tikyld®, Plaquetal®, Ticlodix®), clopidogrel (Plavix®), ticagrelor (Brilique®), triflusal, dypiridamole...
4. Immediately tell the medical team if you are allergic to anything, and if you have a pacemaker/defibrillator device;
5. If you have undergone heart surgeries with valve replacement, or if your cardiologist/surgeon has indicated that you should do antibiotics before some interventions (example: dental repairs) you should immediately tell the medical team about this. However, antibiotic prophylaxis before endoscopy has very exceptional indications.
6. For women under 50 years-old, it is imperative to communicate whether they have any doubt about the possibility of being pregnant;
7. If there are known medical problems that may increase bleeding risk (example: liver cirrhosis, heart problems, blood disorders, kidney function problems), the patient should seek medical advice concerning the procedure indication and safety. Also, the patient should carry updated (less than 3 months) blood test results (blood count with platelet, and coagulation study – INR, prothrombin time).
8. If after carefully reading this document you still have doubts about any aspects, you can seek advice from your Family Doctor, Assistant Physician our within the Gastroenterology department
   You may call _______________ or send an email to _______________. Our team will record your queries and later you will be clarified of your doubts by a Physician or Nurse.

Please, read this entire document carefully. Check that all the information you provided is correct. **Do not hesitate to ask for additional information, and to question the clinical team who requested the exam or is going to perform the procedure – this is a right that assists you.**

If something abnormal is noticed after the patient is discharged, such as fever, thoracic pain, severe abdominal pain, shortness of breath, black stools or difficulty to swallow, the patient should call the Gastroenterology Department or seek the nearest Emergency Department, having its upper gastrointestinal endoscopy report at hand.
