# Supplementary material for: Informed consent in endoscopy: Read, understood, or just signed?
Source: IGIE. 2024 Apr 9;3(2):222–9. doi: 10.1016/j.igie.2024.04.001 (PMC12850725; doi:10.1016/j.igie.2024.04.001)
Supplement: Appendix C — Informed consent form for colonoscopy proposed by the Portuguese Society of Digestive Endoscopy (modified). [file mmc3.pdf]

Colonoscopy with or without sedation (including biopsies and polyp removal as needed) - Free and informed consent

Please, read this entire document carefully as this information is very important. Together with this consent, you will receive a second document with additional information on this procedure.

Colonoscopy is a procedure that allows the observation of the rectum and colon. It is an invasive procedure regardless of whether only diagnostic or therapeutic interventions are performed (biopsies, polyp removal, treatment of bleeding lesions, dilations…). These therapeutic interventions can slightly increase the procedure's risks. Still, colonoscopy is a relatively safe procedure with an overall complication rate of less than 1%. This risk may be higher for elderly people or when there is a medical history of stroke ("thrombosis", "cerebral haemorrhage"), atrial fibrillation (cardiac arrythmia), heart failure or chronic obstructive lung disease.

The most common adverse events include:

- Pain or mid discomfort at the abdomen. This usually improves after the patient releases some bowel gas after the procedure;
- Nausea and/or vomiting;
- Dizziness or fainting sensation after standing at the end of the procedure;
- Headache;
- Pain, redness, and swelling at the puncture site where sedation or medication was administered (in case sedation/medication was administered);
- Muscle pain;
- Allergic reaction to drugs that were administered during the procedure.

Severe complications associated to this procedure are rare, and include:

- **Perforation (rupture)**, of the intestine. This risk is increased in certain groups and situations: age over 75; women; multiple health problems leading to a higher anaesthetic risk; previous abdominal or pelvic surgeries (example: hysterectomy – removal of the uterus) leading to adhesions development ("fixed intestine"); history of abdominal or pelvic radiotherapy; multiple diverticula in the large intestine; inflammatory bowel disease (the risk is higher if there is severe activity at the time of the colonoscopy and/or if the patient is taking corticosteroids); when treatment of lesions/polyps is indicated (the risk is higher if the polyps are large, flat, located in the right side), and when dilation of strictures are performed;
- **Post-polypectomy syndrome**, translating in abdominal pain, fever, signs of peritonitis (abdominal infection);
- **Haemorrhage**, especially if additional procedures are performed (biopsies, polypectomies, dilation…), and if the patient is taking anticoagulant/antiplatelet medication or has low platelets and/or blood clotting disturbances;
- **Cardiovascular and respiratory complications**, such as severe allergic reactions called anaphylaxis, "heart-attack", pulmonary embolism, cardiac arrythmia, stroke, and aspiration of food/fluids into the lungs leading to pneumonia. Though rare, these events are more common during exams where sedation was administered, emergency exams, and for elderly people with other conditions (anaemia, dementia, pulmonary disease, obesity, cardiac insufficiency, valvular heart disease);
- Other extremely rare complications:
  - Related to the bowel preparation – renal failure, dehydration, high blood potassium, pain or abdominal distension, nausea, vomiting, lacerations/erosions of the oesophagus due to vomiting;
  - **Spleen rupture, large abdominal vessel lesions** (mesenteric vessels), **diverticulitis** (diverticula inflammation) and **appendicitis** (ileocecal appendix inflammation). These are very rare, though very serious complications. If you have had previous surgeries do notify your Physician;
  - **Intestinal (colon) explosion**. This is also a rare situation that can occur if the bowel preparation is inadequate and an ignition source (used for polyp removal; argon-plasma coagulation) is used. It is a very serious situation that, in most cases, requires surgical intervention.

Should these complications occur, in most cases they are managed and solved during the endoscopic procedure. However, in certain cases, adequate and definitive complication treatment may require blood transfusion, surgical intervention and hospitalization. If the procedure is scheduled with sedation/anaesthesia, the patient will be monitored throughout the exam. Sedation-related complications may occur as previously discriminated (cardiovascular and respiratory complications, and allergic reactions).

As in other invasive health-related interventions, there is a risk of mortality for both therapeutic and diagnostic procedures, though this risk is extremely low.

The Physician aim is always to perform a complete colonoscopy when this exam is requested. However, this may not be possible due to a variety of situations. Also, colonoscopy is not a 100% proof examination and there is a possibility that some lesions may not be detected (polyps, colorectal cancer) even if the procedure was performed with care and under the best conditions.

Colonoscopy with or without sedation (including biopsies and polyp removal as needed) - Free and informed consent

Do not hesitate to ask for additional information, and to question the clinical team who requested the exam or is going to perform the procedure – this is a right that assists you.

It is essential that you inform the clinical team and the performing physician about your medical history and the medication you usually take. Please pay special attention to the following chart. You must complete this chart with the utmost rigor so that risks are not increased during the procedure.

Please fill in the following table. This is mandatory for the procedure to be performed.

**Please name all the medication you usually take:**		

**Mark with an (X), according to your previous medical history:**		
Previous surgeries?	Yes ( )	No ( )
Hysterectomy (uterus removal)?	Yes ( )	No ( )
Surgeries to the abdomen or pelvis?	Yes ( )	No ( )
If yes, please specify:		
History of diverticula (pouches) in the intestine (colon)?	Yes ( )	No ( )
Chronic lung disease?	Yes ( )	No ( )
Heart disease?	Yes ( )	No ( )
If yes, please specify:		
Pacemaker/defibrillator?	Yes ( )	No ( )
Artificial cardiac valves?	Yes ( )	No ( )
Known medication allergies	Yes ( )	No ( )
If yes, please specify:		
Latex allergy?	Yes ( )	No ( )
Liver cirrhosis?	Yes ( )	No ( )
Blood clotting diseases?	Yes ( )	No ( )
Diabetes mellitus?	Yes ( )	No ( )
Renal disease?	Yes ( )	No ( )
Possible pregnancy?	Yes ( )	No ( )

Please read this entire document carefully, as well as the additional detailed information document that was handed to you. **If you have read the information up to this point, please underline this sentence.** Check that all the information you provided is correct. The doctor performing the procedure will confirm that you are appropriately informed for the procedure to be carried out. If you have no further concerns, please sign the statement below:

I declare that I have been given an informative document, and that I realized the advantages, risks and complications that may be associated with this examination/diagnostic and/or therapeutic intervention (COLONOSCOPY), namely the risk of perforation, haemorrhage, cardiorespiratory complications, including the risk of death, and that I authorize not only its execution, but also the associated procedures and medical acts necessary to resolve possible complications. I have been provided with the information and clarification I considered necessary. I know that I have the right to change my mind by revoking my consent even after signing this document, but I must immediately make it known to the clinical team.

Patient's full name: ____
Date: _____/_____/_____ | Signature of the patient or his/her guardian: __________________________________

DECLARATION

Here in, I confirm that the patient indicated above received the essential information to be adequately informed regarding the intervention that is referred in this document. Additionally, I was available to answer all the questions raised before the procedure.

Physician's full name: ____
Date: _____/_____/_____ | Signature of the performing physician: ______________________________________
