# Supplementary material for: Informed consent in endoscopy: Read, understood, or just signed?
Source: IGIE. 2024 Apr 9;3(2):222–9. doi: 10.1016/j.igie.2024.04.001 (PMC12850725; doi:10.1016/j.igie.2024.04.001)
Supplement: Appendix D — Information leaflet for colonoscopy. Additional information regarding colonoscopy with or without sedation (including biopsy sampling and polyp removal as needed). [file mmc4.pdf]

Colonoscopy with or without sedation (including biopsies and polyp removal as needed) – Additional information

<u>Please, read this entire document carefully as this information is very important – this document complements and details the informed consent form</u>

Colonoscopy is a procedure that allows the observation of the large intestine, which includes the rectum, sigmoid colon, descending colon, transverse colon, ascending colon and the cecum. Sometimes it is necessary to observe the last part of the small intestine named terminal ileum (ileocolonoscopy). When a left colonoscopy is performed, only the rectum, sigmoid colon and descending colon is observed. A flexible device named colonoscope, which is equipped with a small camera that transmits the enlarged images to a monitor within the procedure room, will be introduced through the anus and advanced through the large intestine until it reaches the end of the large intestine, termed the cecum. The procedure will start with the patient lying on its left side with his/her knees bended towards the chest. However, this position may need to be changed during the exam to ease the colonoscope progression. To allow the distension and adequate observation of the bowel wall, air insufflation is necessary, which may cause discomfort/pain (abdominal cramps).

During the procedure, biopsies (small fragments of tissue obtained by a biopsy forceps that runs through the colonoscope) may be required for analysis to complement diagnosis. Also, other instruments may be introduced through the colonoscope to allow diagnostic and/or therapeutic procedures, such as polypectomies (polyps removal with a forceps or snare), endoscopic drug injection, lesion tattooing, and application of therapy/devices aiming for bleeding prevention/control (clips – small pieces of metal; endoloops – ties). Some of these interventions have additional costs, and can be charged after the procedure. Please check this with the health institution where the procedure will take place.

A colonoscopy may last from 10 minutes to one hour, depending on the ease of progression, and the need to perform therapeutic procedures (example: polyp removal).
In some cases, intravenous sedation may be administered and this may significantly decrease the procedure-related discomfort.
After a colonoscopy without intravenous sedation, the recovery is very fast (some minutes). In case sedation is performed, after the procedure the patient will be kept in a surveillance room for about 60 minutes to recover from this sedation, and afterwards, the patient will be discharged but may only leave accompanied, as driving, machine-handling and other high responsibility activities are not allowed for the rest of the day.
On the procedure day, patients most commonly report discomfort due to gas accumulation in the bowel (flatulence, abdominal cramps). This will improve over time as soon as the patient is able to eliminate the accumulated gas. Walking around will ease this process.

In what situations is a colonoscopy indicated?
The recommendation to perform any examination is always made by a Physician according to the patient’s characteristics, complaints and diseases. Also, the decision to perform additional therapeutic interventions will depend on the patient’s clinical situation.

Colonoscopy may be recommended in the following situations:
1. Colorectal cancer screening for the general population and for groups where a higher risk of cancer is recognised (family history of colorectal cancer, inflammatory bowel disease…);
2. Symptom investigation: abdominal pain, digestive bleeding (blood in the stools), changes in the intestinal transit pattern;
3. Diagnosis: anaemia and diarrhoea causes, inflamed mucosa biopsies, tumour detection;
4. To clarify/proceed with the treatment of previous endoscopic findings, or doubts raised in different exams (radiographies, abdominal or pelvic computed tomography, ultrasounds or blood results);
5. Treatment: colonoscopy may be a solely diagnostic procedure, but may also be therapeutic and curative allowing for polyp removal, intestinal dilation, foreign body removal, and application of therapy aiming for bleeding prevention (endoscopic drug injection, application of thermal energy on anomalous vessels…).
   * It should be noted that the decision to perform a specific therapy will depend on the clinical situation and circumstance: bulky, flat or multiple polyps, unstable colonoscope position, etc… may require the intervention to be postponed or performed under different conditions.

Colonoscopy with or without sedation (including biopsies and polyp removal as needed) – Additional information

Are there alternative procedures to colonoscopy?
Depending on the exam indication and aim, there may be alternatives that you should discuss with your Physician, such as tomography assisted colonography ("virtual colonoscopy"), capsule colonoscopy, and faecal blood testing (the latter is only indicated for colorectal cancer screening in asymptomatic individuals with no family history of colorectal cancer). However, these alternatives are not indicated in all circumstances, have several limitations compared to colonoscopy, and are not without risks.

It matters to state that not taking a colonoscopy that was advised by a Physician may be associated with the absence/delay of diagnosis and/or treatment of lesions and pathologies affecting the rectum, the colon and the terminal ileum (example: colorectal cancer and polyps' treatment, which can be curative). If you have any doubts regarding your indication to undergo this exam, you can seek advice from your Physician. You will be given the opportunity to speak with the performing Physician and with the Anaesthesiologist (in case your exam is scheduled under deep sedation performed by an Anaesthesiologist) before the colonoscopy is carried out.

Is the colonoscopy going to be total? Not always.
The Physician's aim is always to perform a complete colonoscopy, and observe the ileocecal valve and the appendix orifice, when a complete colonoscopy is requested. However, this may not be possible due to a variety of situations: if the bowel preparation is inadequate or if the colonoscope progression turns out to be very difficult, the Physician may decide to interrupt the exam to ensure the patient's safety.

Colonoscopy is not a foolproof examination and there is a possibility that some lesions may not be detected (polyps – 2 to 26%, colorectal cancer – 3 to 6%) even if the procedure was performed with care and under the best conditions. **Also, polyp removal may decrease the risk to develop colorectal cancer, but undergoing a colonoscopy does not give you an absolute protection and patients may still have a tumour after a colonoscopy. This risk increases if the bowel preparation is not appropriate. Therefore, patients must strictly follow the instructions given for that purpose.**

During colonoscopy, endoscopic images will be taken for photo documentation, unless there is a technical problem that does not allow it.

Is the colonoscopy risk-free. No.
This procedure is invasive regardless of whether only diagnostic or therapeutic interventions are performed (biopsies, polyp removal, treatment of bleeding lesions, dilations…). These therapeutic interventions can slightly increase the procedure risks. Still, colonoscopy is a relatively safe procedure with an overall complication rate of less than 1%. This risk may be higher for elderly people or when there is a medical history of stroke ("thrombosis", "cerebral haemorrhage"), atrial fibrillation (cardiac arrythmia), heart failure or chronic obstructive lung disease.

The most common adverse events include:
- Pain or mid discomfort at the abdominal level. This usually improves after the patient releases some bowel gas after the procedure;
- Nausea and/or vomiting;
- Dizziness or fainting sensation after standing at the end of the procedure;
- Headache;
- Pain, redness, and swelling at the puncture site where sedation or medication was administered (in case sedation/medication was administered);
- Muscle pain;
- Allergic reaction to drugs that were administered during the procedure.

Severe complications associated to this procedure are rare, and include:
- **Perforation (rupture)** of the intestine. This risk is increased in certain groups and situations: age over 75; women; multiple health problems leading to a higher anaesthetic risk; previous abdominal or pelvic surgeries (example: hysterectomy – removal of the uterus) leading to adhesions development

("fixed intestine"); history of abdominal or pelvic radiotherapy; multiple diverticula in the large intestine; inflammatory bowel disease (the risk is higher if there is severe activity at the time of the colonoscopy and/or if the patient is taking corticosteroids); when treatment of lesions/polyps is indicated (the risk is higher if the polyps are large, flat, located in the right side), and when dilation of strictures are performed;

- **Post-polypectomy syndrome**, translating in abdominal pain, fever, signs of peritonitis (abdominal infection);
- **Haemorrhage**, especially if additional procedures are performed (biopsies, polypectomies, dilation…), and if the patient is taking anticoagulant/antiplatelet medication or has low platelets and/or blood clotting disturbances;
- **Cardiovascular and respiratory complications**, such as severe allergic reactions called anaphylaxis, "heart-attack", pulmonary embolism, cardiac arrythmia, stroke, and aspiration of food/fluids into the lungs leading to pneumonia. Though rare, these events are more common during exams where sedation was administered, emergency exams, and for elderly people with other conditions (anaemia, dementia, pulmonary disease, obesity, cardiac insufficiency, valvular heart disease);
- Other extremely rare complications:
  - Related to the bowel preparation – renal failure, dehydration, high blood potassium, pain or abdominal distension, nausea, vomiting, lacerations/erosions of the oesophagus due to vomiting;
  - **Spleen rupture, large abdominal vessel lesions** (mesenteric vessels), **diverticulitis** (diverticula inflammation) and **appendicitis** (ileocecal appendix inflammation). These are very rare, though very serious complications. If you have had previous surgeries do notify your Physician;
  - **Intestinal (colon) explosion**. This is also a rare situation that can occur if the bowel preparation is inadequate and an ignition source (used for polyp removal; argon-plasma coagulation) is used. It is a very serious situation that, in most cases, requires surgical intervention.

Should these complications occur, in most cases they are managed and solved during the endoscopic procedure. However, in certain cases, adequate and definitive complication treatment may require blood transfusion, surgical intervention and hospitalization. If the procedure is scheduled with sedation/anaesthesia, the patient will be monitored throughout the exam. Sedation-related complications may occur as previously discriminated (cardiovascular and respiratory complications, and allergic reactions).

As in other invasive health-related interventions, there is a risk of mortality for both therapeutic and diagnostic procedures, though this risk is extremely low.

Therefore, it is important that you take some precautions to ensure that the exam runs as easy as possible and that risks are lowered:

1. Do strictly comply with the bowel preparation regime and scheme;
2. If your exam is scheduled to be performed under sedation, you must strictly comply with the fasting period recommended in the respective bowel preparation schema. After procedures with sedation, the patient will be discharged but may only leave accompanied, as driving, machine-handling and other high responsibility activities are not allowed for the rest of the day. Also, within 12-24h after the exam, the patient must not sign legal binding documents. If the patient is not accompanied, the procedure must not be performed with sedation and in some cases, it may even be cancelled;
3. The patient must bring a record of all medication he/she usually takes, and write it down in the informed consent table where this is requested;
4. Immediately tell the medical team if you are allergic to anything, and if you have a pacemaker/defibrillator device;
5. If you have undergone heart surgeries with valve replacement, or if your cardiologist/surgeon has indicated that you should do antibiotics before some interventions (example: dental repairs) you should immediately tell the medical team about this. However, antibiotic prophylaxis before colonoscopy has very exceptional indications.

6. For women under 50 years-old, it is imperative to communicate whether they have any doubt about the possibility of being pregnant. **Colonoscopy may be unadvised in this setting**;

Colonoscopy with or without sedation (including biopsies and polyp removal as needed) – Additional information

7. In case you are takin antiplatelet medication:
   - You may keep taking acetylsalicylic acid, unless you are specifically told otherwise - (Aspirina®, AAS®, Cartia®, Tromalyt®)
   - Antiplatelets to be stopped 5 days before the colonoscopy are: clopidogrel (Plavix®), prasugrel (Efient®), ticagrelor (Brilique®)
   - Antiplatelets to be stopped 10 days before the colonoscopy are: ticlopidine (Tiklyd®, Plaquetal®, Ticlodix®)
   - In case you ever had a myocardial infarction (“heart attack”), coronary stents placed or have had a stroke, the suspension/replacement of these medications should be previously discussed with your attending Physician.
8. Anticoagulant medication should be stopped 5 days before colonoscopy – warfarin (Varfine®), acenocumarol (Sintron®), fluindione). However, discuss this with your attending Physician, and do not stop these medications on your own as it may have to be replaced by another. Also, you will have to do a blood test termed “INR” the day before or on the procedure day. These recommendations are different for the new anticoagulants – Pradaxa®, Xarelto®, Eliquis®, Lixiana®. Please discuss this with your attending Physician, as you should receive specific instructions regarding these medications.
9. The resumption of any medication that was stopped should be done according to the performing Physician recommendations;
10. If there are known medical problems that may increase bleeding risk (example: liver cirrhosis, heart problems, blood disorders, kidney function problems), the patient should seek medical advice concerning the procedure indication and safety. Also, the patient should carry updated (less than 3 months) blood test results (blood count with platelet, and coagulation study – INR, prothrombin).
11. If after carefully reading this document you still have doubts about any aspects, you can seek advice from your Family Doctor, Assistant Physician or within the Gastroenterology department
    You may call _______________ or send an email to _______________. Our team will record your queries and later you will be clarified of your doubts by a Physician or Nurse.

Please, read this entire document carefully. Check that all the information you provided is correct. **Do not hesitate to ask for additional information, and to question the clinical team who requested the exam or is going to perform the procedure – this is a right that assists you.**

If something abnormal is noticed after the patient is discharged, such as fever, thoracic pain, severe abdominal pain, shortness of breath, black stools or difficulty to swallow, the patient should call the Gastroenterology Department or seek the nearest Emergency Department, having its colonoscopy report at hand.
