# Supplementary material for: Informed consent in endoscopy: Read, understood, or just signed?
Source: IGIE. 2024 Apr 9;3(2):222–9. doi: 10.1016/j.igie.2024.04.001 (PMC12850725; doi:10.1016/j.igie.2024.04.001)
Supplement: Appendix E — Questionnaire on informed consent for endoscopic procedures applied before the endoscopic procedures. [file mmc5.pdf]

SNS SERVIÇO NACIONAL DE SAÚDE

CENTRO HOSPITALAR TONDELA | VISEU, EPE

ID

Questionnaire on informed consent for endoscopic procedures

1. Gender ____ male ____ female

2. Age ____ years

3. Education level

____ uneducated (does not read or write)

____ uneducated (reads or writes)

____ primary education

____ lower secondary education

____ upper secondary education

____ post-secondary non-tertiary education

____ short-cycle tertiary education

____ bachelor's or equivalent

____ master's or equivalent

____ doctorate or equivalent

____ does not answer

4. Endoscopic procedure performed ____ upper gastrointestinal endoscopy ____ colonoscopy

4.1 Conscious sedation ____ without sedation ____ with sedation

5. Previous endoscopic procedure ____ yes ____ no ____ does not answer

5.1 If yes, where ____ CHTV ____ other ____ does not answer

EVALUATE THE READING OF THE INFORMED CONSENT FORM

6. Has the patient read the informed consent form ____ yes ____ no ____ does not answer

If he had read:

6.1 Did he had doubts ____ yes ____ no ____ does not answer

6.1.1 If yes, what doubts

____ regarding the procedure itself
____ risks and complications
____ why he needs the procedure
____ benefits/disadvantages of the procedure
____ alternative exams to the procedure
____ regarding conscious sedation
____ regarding bowel preparation
____ does not answer

6.2 Did he changed his mind regarding the procedure ____ yes ____ no ____ does not answer

If he had not read:

6.3 Reason for not reading

_____ ignored/did not want to read
____ no time for reading
____ did not understand and stop reading
____ was not told to read
____ afraid of reading
____ uneducated (does not read)
____ does not answer

EVALUATE IF ADEQUATE READING OF THE INFORMED CONSENT FORM

7. Criteria for adequate reading

**Signature of the form**	____ yes	____ no
**Filling of the table questionnaire**	____ yes	____ no
**Performance of the text instruction**	____ yes	____ no
